# My life with primates

**DOI:** 10.1007/s10329-022-01025-w

**Published:** 2022-10-15

**Authors:** Vernon Reynolds

**Affiliations:** 1 School of Anthropology, Oxford University, Oxford, UK; 2 Budongo Conservation Field Station, Masindi, Uganda

**Keywords:** Chimpanzee, Budongo Forest, Uganda, Field station, Field research, Conservation

## Abstract

In this paper I recall some of the significant moments of my career as a primatologist, including some of the intellectual conflicts I encountered between anthropology, sociology and zoology. From an initial interest in ethics and evolution, I undertook research on rhesus monkeys in captivity and then on chimpanzees in the wild. Influenced by Japanese primatology as well as Western approaches, this led to my work on the problems of describing primate behaviour, but this more theoretical approach was superseded by empirical work embodied in the founding of the Budongo Conservation Field Station. I describe the initial creation of the field station in 1990 and some of the research directions we have followed since that time. The paper ends with a focus on conservation, this being of increasing importance as the Budongo Forest faces ever increasing threats from industry.

## Introduction

**Fig. 1:**
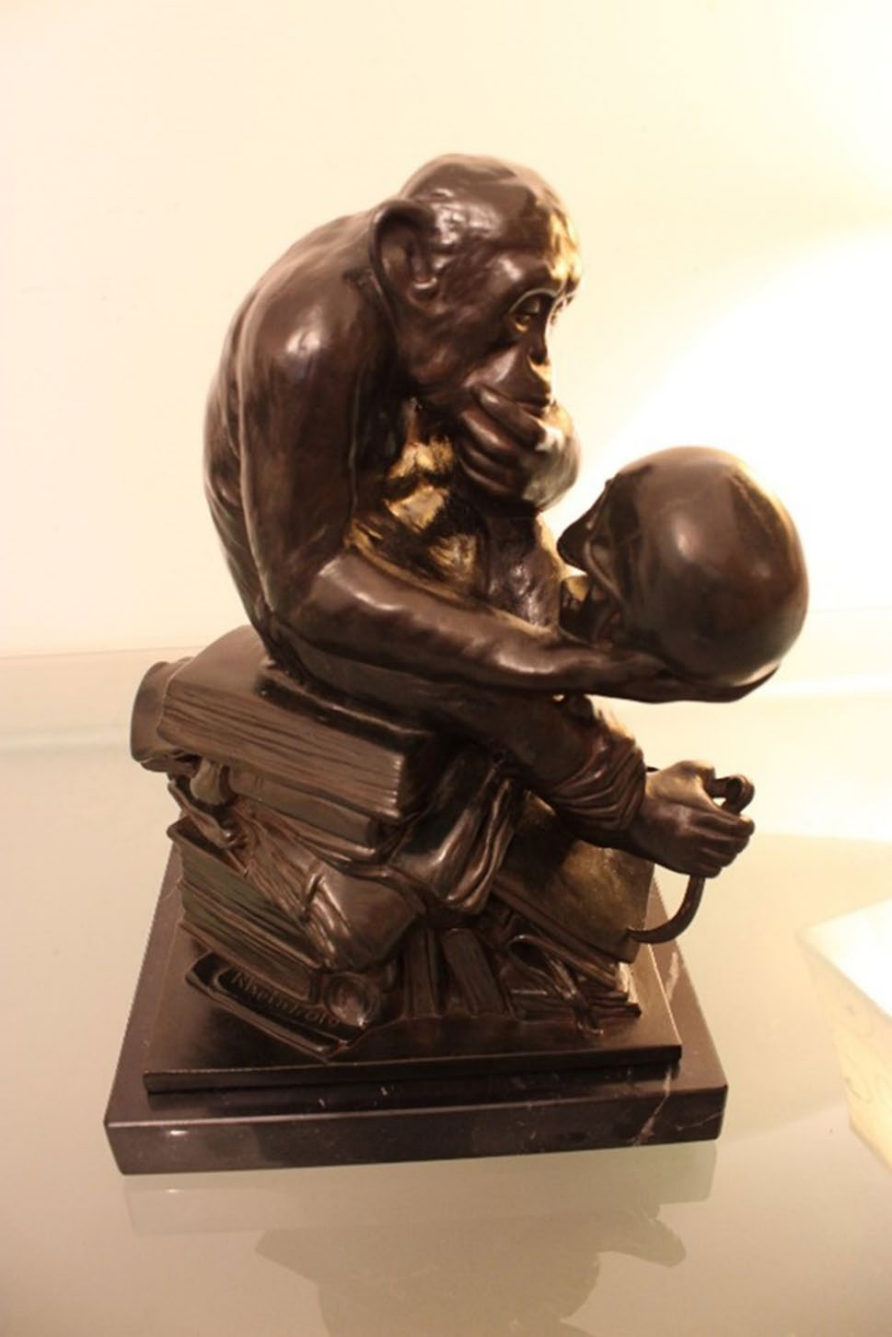
Ape with human skull. Bronze statuette by Hugo Rheinhold

On a shelf in my study is a bronze statuette (Fig. 1). It shows a chimpanzee, but this is no ordinary chimpanzee. He sits on a pile of books, one hand covering his mouth in contemplation, while the other hand holds a human skull. He gazes down at the skull with evident interest. One of the books beneath him is titled “*DARWIN*”. In his left foot he holds a pair of callipers, and beneath the foot is another book, a Bible, open at a page where we see inscribed the words “Eritis sicut Deus” (“You shall be as God”), the first part of a passage that continues “knowing good and evil...”.

This statuette was created by my great-uncle Hugo Rheinhold, who died in 1900. He saw the chimp in the Berlin Zoo and, drawing on his philosophical ideas, he sculpted it. Science and ethics, evolution, intelligence and morality—all are represented in this one piece. It has since been copied innumerable times. For present purposes the question is: did Hugo’s masterpiece start me off on the road to primatology? And the answer is: I don’t know. I first saw it as a teenager and maybe it left its mark, but we will never know.

I was born into a German family and brought up in England. At the age of 18 I was called up to do 2 years of national service (1954–1956). While in the army, a friend suggested I might like to go to university and study anthropology. This I did. From 1956 to 1959 I studied anthropology at University College London, and during this time was taught social and physical anthropology, and archaeology. Physical anthropology included Darwinian evolution and man’s relationship to other primate species. I decided to study the social life of a colony of rhesus monkeys living at Whipsnade Zoo, the field outpost of London Zoo. Having obtained permission, I embarked on a study which led to a thesis entitled “The social life of a colony of rhesus monkeys (*Macaca mulatta*)”. The thesis described the social behaviour of a group of captive rhesus monkeys that I observed over a 10-month period. I described the dominance relations between males and between females, and also grooming and sexual behaviour. I also described triad behaviour patterns in which three monkeys interacted simultaneously, as for instance when one monkey redirected its aggression from the more dominant monkey to the third, less dominant one. The thesis was accepted for the degree of doctor of philosophy (Reynolds 1961).

During this time I encountered the work of Japanese primatologists Kinji Imanishi, Masao Kawai, Junichiro Itani, Shunzo Kawamura and Yukimaru Sugiyama. Their work provided a perspective and added insights into the importance of kinship for determining social status within the group that I could not get from my study of the zoo group. I understood then the importance of fieldwork and the need to study primates in the wild.

Following the rhesus study I obtained a small grant from London University which enabled my wife Frankie and me to go to Africa for a year to study the social life of wild chimpanzees. Chimpanzees are closely related to humans, and as an anthropologist I was on the lookout for insights into human behaviour. Chimpanzees are closer to our pre-human ancestors than other primate species. Hence, just as great-uncle Hugo had done, I made the decision to focus on chimpanzees, a decision that has guided me throughout my academic life.

## Travel to Africa

In 1962 Frankie and I travelled to Africa. We chose Uganda as we had heard that chimpanzees lived in the forests in the west of the country. We had decided to study forest-living chimpanzees because the forests of Africa were thought to be the habitat in which our ape ancestors evolved, and which the early hominins gradually exchanged for more open woodland environments, and also for savannah and lakeside habitats.

Arriving at Makerere University, Kampala, we had a big surprise. Our field supervisor, Prof. Nils Bolwig, had just left Uganda for the University of Lagos, in Nigeria. I needed a supervisor in order to comply with the conditions of the grant. Fortunately one of the members of staff at the nearby Virus Research Institute in Entebbe, Dr Alec Haddow, had himself done primate fieldwork, and he stepped in to supervise our work.

**Fig. 2:**
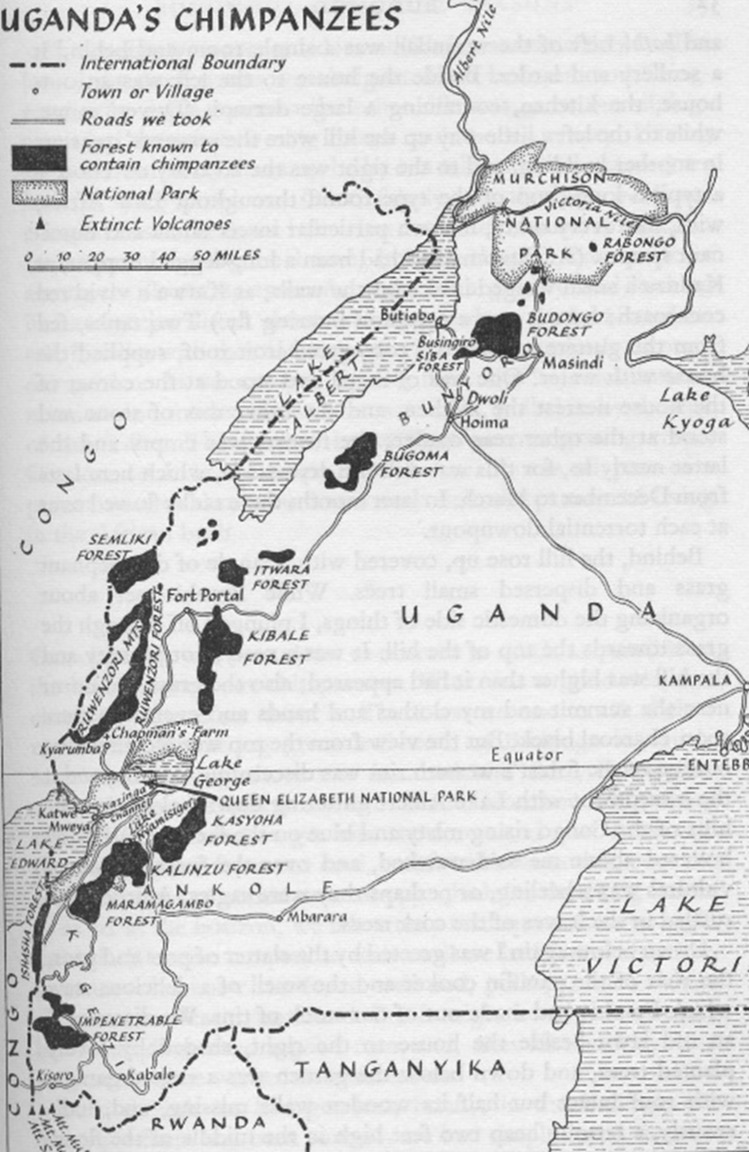
Map of Ugandan forests inhabited by chimpanzees

We obtained an old Land Rover and travelled to the west of the country (Fig. 2). Beginning with the Budongo Forest, we travelled south, visiting the various forests along the way: Bugoma, Semliki, Ruwenzori, Kibale, Kalinzu and finally the Impenetrable Forest, now known as Bwindi. Our first journey to western Uganda took place over a period of 1 month, during which time we spent 3–5 days in each of the western forests.

My first book “*Budongo: a Forest and its Chimpanzees*” (1965) includes a description of our journey. We glimpsed chimpanzees, or heard them, in all the forests we visited. We decided to focus on Budongo Forest, which had the advantage that it was home to a large sawmill, Budongo Sawmills, which kindly gave us access to their telephone and mail facilities. The then manager and other members of staff at the mill were very welcoming. So, after our first month in Uganda, we returned to Budongo Forest.

Thus began our first field study of chimpanzees, which lasted 9 months. We had been given the use of a small Forest Department rest house located on Busingiro Hill just outside the forest to the southwest. This was an ideal location for us from which to enter the forest on a daily basis.

## Field study in the forest

We obtained the services of a tracker, Manueri, who had been working for the sawmill and thus knew the forest. Day by day we entered the forest, then sat and waited for the sound of chimpanzees calling. As soon as we heard calls, we set off with compass and notebook to see if we could find the chimpanzees. Many days we scarcely saw them at all, but sometimes we had productive encounters. We took extensive notes and began to build up a picture of their lives, their behaviours, and their social organisation. To our surprise, groups of chimpanzees formed and re-formed each day, with different individuals joining and leaving, moving from one group to another along the forest floor. The current term for this form of social organisation, fission–fusion, describes very well the movement pattern of chimpanzees in a community. We noted that larger groups formed when food was plentiful or concentrated, whereas smaller groups were associated with a shortage of food or when food was widely dispersed. We did not know that chimpanzee males stay in their natal group whereas females mostly emigrate to a new community on reaching adolescence: that discovery came later. Also, we did not use the word “community” for the chimpanzees living in the same area, although it is a good term as it distinguishes chimpanzees from other primate species. We did not see any kind of tool-making involving sticks or stones, but during our 9-month study we did observe chimpanzees making leaf sponges and dipping them into water, mainly in tree boles, for drinking by squeezing it out into their mouths. Indeed, to this day, stick and stone tool-making and use is very limited in the Budongo chimpanzees, in contrast to chimpanzees at other sites in East Africa, which use sticks or grass stems to extract termites from their mounds. This behaviour does not happen at Budongo. Nor did we see the use of stone tools as observed in West African chimpanzees, which use stones to crack open hard nuts.

What might be the reason for this lack of technology in the Budongo chimpanzees? I have often wondered about this, but no explanation has so far been found. However, there are several possible explanations. From our knowledge of chimpanzee cultures (Whiten et al. 2000, 2001) it may be that tool use and tool-making simply have not reached Budongo. Alternatively, it is possible that the Budongo chimpanzees have never needed to use tools other than in terms of their very limited use that we see today. For example, there are no tree species in Budongo Forest that produce hard nuts, and termite mounds inside the forest are less amenable to the use of grass stems or “wands” to collect the insects than the *Macrotermes* mounds outside the forest.

Due to limited time and resources, we were not able to cut trails into the forest, but rather just followed the chimpanzee hoots, with Manueri slashing a path for us through the understorey with his panga, which was a difficult way of moving through dense undergrowth, especially when we encountered spiky rattan creepers in swamp forest.

## Return to the UK

Returning to the UK heralded a difficult time. My wife and I had very little money. We lived in an attic flat in London, and the winter of 1962–1963 was one of the coldest on record in the UK. We settled down to write, and to find jobs. Before long we received an invitation from Stanford University in California to join a group of field primatologists to contribute to a book, the first ever on this new area of study. It was edited by Irven deVore and titled “*Primate Behavior: Field Studies of Monkeys and Apes*” (DeVore 1965). We wrote a chapter in the book, detailing for the first time the social organisation and behaviour of forest-living chimpanzees.

We spent 6 months in 1963 on that book project, at Stanford’s Center for Advanced Studies in the Behavioral Sciences. It was a charmed life, but that ended when we returned to the UK, where we still had no jobs. I applied for a post at my alma mater, University College London, but the Anthropology Department there did not teach primatology; indeed, the subject was unknown in the UK at the time. Eventually I found a job with a magazine called “*Animals*”, writing articles for them on African wildlife.

At the end of 1964, out of the blue, came another unexpected invitation. My wife and I were invited to New Mexico, to the 6571st Aeromedical Research Laboratory at Holloman Air Force Base in Alamagordo, New Mexico, a facility managed by the National Aeronautics and Space Administration. The invitation was to observe the chimpanzees that were being kept there in readiness for space flight, to ascertain whether they were “normal” after their prolonged period in captivity. We were asked to look at the Holloman chimpanzees to ascertain whether any of them were unsuitable for Aerospace research because they had behavioural disorders. This we did to the best of our abilities. The main difference we found between the chimpanzees at Holloman and those in the wild was that the former were unable to split up and form separate groups. As a result, the level of aggression between the Holloman chimpanzees was higher, leading on one occasion to one subadult male leaping into the moat surrounding the enclosure. It would have drowned if I had not gone into the moat and pulled it out, whereupon it was resuscitated and thence removed from the colony.

Back in the UK, I was invited to write a book about the apes—the gibbon, orangutan, chimpanzee and gorilla—by the publisher Edward Payson Dutton, based in New York. I settled down to do this, working in the library of the British Museum which, being the UK’s national library, contained all the books I needed to research the subject. Titled “*The Apes*” (Reynolds 1967), the book was enjoyable enough to write, yet I realised that my real interest lay in understanding chimpanzee behaviour and not in writing books. However, time and money were not yet available for a return to Uganda.

## Japanese primatology

Reading primate journals led me to the Japanese journal “*Primates*”. I read a paper in it by Yukimaru Sugiyama (1968), about his studies on the Budongo chimpanzees. He worked in the same area where my wife and I had undertaken research, but he had gone beyond what we had achieved by being able to recognise many of the individuals in the Busingiro community, and then naming them. In most other respects his studies confirmed our own findings. After our study and that of Sugiyama, another study was undertaken a few years later, by Akira Suzuki (1971). Suzuki was the first to witness and photograph chimpanzee infanticide. The photos in his paper were of good quality and proved something unknown at that time: that an adult male chimpanzee could kill an infant of his own species and eat it. The paper was ignored by the scientific community because the whole idea of chimpanzee infanticide was new, and its occurrence was thought to be an aberration, i.e. the male in the photographs was in some way pathological and not “normal”. Science can be slow and unwilling to accept new ideas.

There was a similar response to the findings of Sugiyama (1965), as described in his paper in this series (Sugiyama 2022), who also observed infanticide, in Hanuman langurs. As with the findings of Suzuki (1971), this behaviour was thought by the scientific community to be a kind of pathology, and Sugiyama’s work was not fully understood. He showed that infanticide in langurs happens when a new male takes over a one-male group. The underlying mechanism for this was not understood by zoologists at the time, it could not be explained by conventional Darwinian theory, and so it was rejected for years, before gaining acceptance later on.

I myself had a somewhat similar experience some years later, when I was at Oxford University. In our initial chapter in DeVore (1965), and in my book (Reynolds 1965), my wife and I had described the pant-hoot chorusing of chimpanzees, which we called (using the Swahili word)* kelele*. We had noted that when large groups of chimpanzees collected on a fig tree with ripe fruit, they joined together to produce loud group calls, which travelled far across the forest canopy. We also noticed that sometimes the calling was responded to by other chimpanzees across the trees. And at times the responding chimpanzees arrived at the tree shortly after the calling and joined those already there. It seemed clear enough to us that chimp groups called to other groups announcing the whereabouts of good food, and inviting others to come and join them. However, when I mentioned this in a talk to the Animal Behaviour Group at the Zoology Department at Oxford, I was informed (after the talk) that I must be mistaken. Animals, I was told, do not announce the whereabouts of good food, they keep it to themselves, selection operates at the level of the individual, not the group. Some years later the theory of kin selection, which had been proposed by Hamilton (1964), became more widely accepted and was able to explain the chimpanzees’ *keleles*: the males in the groups of chimpanzees that called to each other were closely related, and so their genetic interests, as we now know, were served by their co-operation.

## Bristol University

My first university job came in 1966, in the Department of Sociology at Bristol University. I spent 6 years there teaching anthropology. It was enjoyable, but I met a challenge from an unexpected quarter. The period around 1968 was a time of political ferment in Europe, with talk of revolution in France and Germany and student riots that spread to England. Bristol University students rioted, and some members of staff of the Sociology Department were involved. These same people challenged me to stop lecturing about the evolution of human behaviour. I was told that such lectures were very right-wing and against the principles the students were rioting for. The reason my lectures on evolution were perceived as right-wing was explained to me by a Marxist colleague, who told me that the main point of studying humanity was to change it, not to see it as equivalent to animal instinct. He explained that by seeing human behaviour as a product of evolution I was denying the possibility of radical change, a right-wing position. I was shocked. These political attacks, coming from colleagues who were otherwise friendly and reasonable, came as a bitter blow. However, I did not stop teaching my subject—human behavioural evolution. Fortunately for me, academic freedom was on my side.

Just as my time at the Anthropology Department at University College London had taught me how to respond to Christian objections to Darwinian ideas, so my time at the Department of Sociology at Bristol taught me that it was a mistake to challenge Darwinian biology with Marxist sociology. Religion, evolution, and revolution belong in different worlds of ideas, with different relevance to the human condition. To set them up as opposites is a mistake.

I learned much from the Department of Sociology at Bristol University. I learned that overarching our biological inheritance and behavioural tendencies is a cognitive world of complex constructs that constitutes our human cultures. These constructs act as absolute dos and don’ts for our actions in everyday life. The work of Erving Goffman influenced me. His book “*The Presentation of Self in Everyday Life*” (Goffman 1959) was an eye-opener because I had not previously understood the extent to which human behaviour was determined by the cultural forces around us. I would never again be able to move easily, i.e. make straightforward comparisons, between non-human primate behaviour and human behaviour, which I later came to call “action” in my book “*The Biology of Human Action*” (Reynolds 1976).

## Oxford University

In 1971, while working at Bristol University, a lectureship came up at Oxford University in the Department of Physical (later Biological) Anthropology. I applied and was fortunate to be offered the position. Thus began my time at Oxford, where I remained for the rest of my career. My first reaction on arriving at Oxford was one of relief, as I could continue teaching my preferred subject without being accused of right-wing sympathies. I joined in with the work of the department and continued teaching primate behaviour and human evolution. A new degree at Oxford was just starting when I arrived, B.Sc. Human Sciences, and I very much liked the idea that this degree extended its reach from biology to sociology. I enjoyed teaching human sciences students, and some of those I taught have remained friends to this day.

While at Oxford I undertook various kinds of research as well as teaching. A colleague and I wrote on the ecology of religion, a subject that had interested me since childhood (Reynolds and Tanner 1983). Some years later this theme came up again, and a colleague and I edited a book that explored the extent to which religions acted as an independent force in cultural evolution, and to what extent they were shaped by ecological and economic forces (Jones and Reynolds 1995).

Meanwhile Uganda had been taken over in 1971 by Idi Amin, who soon proved to be a murderous dictator, making any return to further field work in Budongo very unwise. Tanzania was calm, however, and so in 1977 I made a visit to the Japanese research site at Mahale. I was accompanied on this trip by my colleague Yukimaru Sugiyama, and we were able to observe the Mahale chimpanzees at fairly close quarters. The Japanese researchers provisioned their study subjects with sugarcane. My co-workers and I had never done any provisioning in Budongo during our time there, but at Mahale I was able to see the advantages of provisioning. The chimpanzees could be observed from closer quarters and were not so scared of people. I understood that this was how Japanese researchers were able to identify individuals. Naming and provisioning of primates was in fact first done by Japanese researchers in the 1950s, in their studies of Japanese macaques. This method was now successfully used in Tanzania, and had initially been used by Jane Goodall at Gombe Stream Reserve. However, provisioning involves interference with the normal feeding ecology of wild animals, and despite the advantages of provisioning in speeding up the process of habituation, it distorts their natural foraging behaviour. When we returned to Budongo some years later, we did not use provisioning as a research method, and all the behaviour recorded at Budongo from 1990 to the present day has been based on minimising interference from researchers.

At Oxford I embarked on a new kind of fieldwork: the study of human beings living normal lives not far from Oxford. Under the supervision of my head of department, Geoffrey Harrison, and together with a number of colleagues in the department, we made a number of studies of the biological characteristics of the inhabitants of a village to the north of Oxford, such as their urinary corticosteroid levels in relation to their lifestyles (Harrison 1995).

Also while at Oxford a colleague, Rom Harré, and I discussed a topic that had puzzled me since the days of my study of rhesus monkeys: the way in which we human beings describe primate behaviour, applying human words to describe non-human species. One of my research students, Pamela Asquith, made a close study of anthropomorphism in primatology (Asquith 1981). Our discussions led to a conference at Bad Homburg in Germany, to which we invited a number of primate fieldworkers, anthropologists and linguists. The outcome of the conference was published as a book, “*The Meaning of Primate Signals*” (Harré and Reynolds 1984). In it we focused on how primate behaviour has been described and understood, and to what extent anthropomorphism and linguistic biases might influence how we humans understand the behaviour of non-human primates. In particular, the use of human language inclines us to understand primate behaviour in the same terms as we understand the words we use, and anthropomorphism inclines us, rightly or wrongly, to impute similar feelings and emotions to primates as to ourselves.

In 1974, soon after my job at Oxford began, I went to Japan for the first time, to attend a congress of the International Primatological Society (IPS), held at Nagoya. This was my first IPS meeting and I much enjoyed it. I have remained a member of IPS since 1974, and attended many of their meetings, including a second one in Japan in 1990. At this later meeting I travelled with my colleague and friend the late Duane Quiatt, who had come to Budongo a number of times. In 1990 my wife and I visited Duane in his home in Boulder, Colorado, where together we worked on a book we had started the year before when Duane came as a research fellow to Magdalen College, Oxford. The book was titled “*Primate Behaviour: Information, Social Knowledge, and the Evolution of Culture*” (Quiatt and Reynolds 1993).

At the 1990 IPS Congress in Kyoto, where I presented a paper on the study of kinship by Japanese primatologists, I mentioned that Western zoologists had had considerable difficulty in accepting the possibility that non-human primates could recognise kinship. That difficulty had by then largely been overcome, but there was still much resistance among social anthropologists to the idea that kinship in non-human primates was akin to kinship in human societies. Back at Oxford, my colleague Nick Allen and I discussed the similarities and differences between monkey and human kinship. We discussed whether human kinship might have evolutionary origins, or whether the principles of human kinship were invented as a way of organising and classifying society.

One aspect of this debate concerns the distinction between mating and marriage. In due course I became involved in a conference on this subject, and I co-edited a book with the title “*Mating and Marriage*” (Reynolds and Kellett 1991). Eventually I came to realise that the term “kinship” as used by social anthropologists implied a system of “marriage”, so that without the latter there could be no kinship. This of course was very different from the meaning of kinship in primatology, where it was determined by mating and the production of offspring without the intervention of a marriage ceremony. This conference led me to understand the value of scientific discussions, open-mindedness, and interdisciplinary exchange in promoting research and ideas.

All this time I had been following events in Uganda, where with Amin deposed, civil war had broken out, and was now nearing its end. In 1986 Yoweri Museveni was able to defeat the dictatorship that had run Uganda for 15 years, and to establish a fairer and more democratic form of government. This was the moment I had been waiting for, and I wasted no time in planning a return to Uganda. I had read a newspaper article from the New Vision, Uganda’s leading paper, in 1988, sent to me by Shirley McGreal of the International Primate Protection League, which was based in South Carolina. This article featured two young chimpanzees that had been confiscated at Entebbe Airport. The headline on the front page of the paper was, in large print, “*CHIMP RACKET BLOWN*”. The article gave details of the poaching of young chimpanzees from the forests of Uganda, including Budongo. The chimpanzees, some less than a year old, were bundled into the back of vehicles and taken to Entebbe Airport, from where they were flown to Dubai and other destinations to be pets for wealthy people.

## A field station?

When I read this report in 1988, I realised that a short field study would not be the best way to approach this problem of poaching. Something more long-lasting and permanent was needed. That was when I started thinking about establishing a field station, something I had never imagined before. To do that would require a lot of forward planning and searching for funds. So I set to work writing to all the conservation agencies I could think of. I found that the larger ones had already committed themselves to supporting various projects into the future and did not have any money to start new projects. Fortunately some smaller agencies did provide me with a number of small grants, which were extremely useful in getting things off the ground.

In Kampala I stayed at the home of Derek Pomeroy, who kindly advised me on all the permissions I would need and how to get them. Derek also introduced me to a Ugandan student, Chris Bakuneeta, who had recently completed his M.Sc. in zoology and was looking for a job. I worked closely with Chris, who is a strong and cheerful person, for many years.

Our first job was to establish whether there were any surviving chimpanzees in the Budongo Forest. Chris knew that all the elephants that used to live in the forest had been killed, for food and ivory, by soldiers during the civil war. Had the same happened to the chimpanzees? We drove to Budongo and stayed at the guest house of the nearby Nyabyeya Forestry College, located just outside the forest. There was a rainwater tank up at the abandoned sawmill site where we went to draw water. One day, while we were filling up our jerrycans, we heard chimpanzees calling from the surrounding trees, so at least we knew chimpanzees were still present in the forest. We moved towards the sounds and saw a mother and her infant. They immediately ran away and we realised that they were very afraid of us, which was hardly surprising since chimpanzees had been shot at and their infants taken by poachers for the past 15 years. This reaction was even more extreme than the shyness the chimpanzees had shown during our first visit in 1962.

I returned to the UK after that first visit in the spring of 1990, leaving Chris to begin the work of setting up a field station. His mandate was to find six good field assistants whose job it would be to go into the forest daily, searching for chimpanzees and gradually attempting to habituate them to human presence. At the same time Chris would find some local men to cut trails in the forest on a north—south, east—west grid pattern. Chris and I communicated by email and he informed me he had succeeded in finding some good field assistants, and also some good trail cutters. At that time the project was still based at the college guest house.

## Renovation, habituation and research

**Fig. 3:**
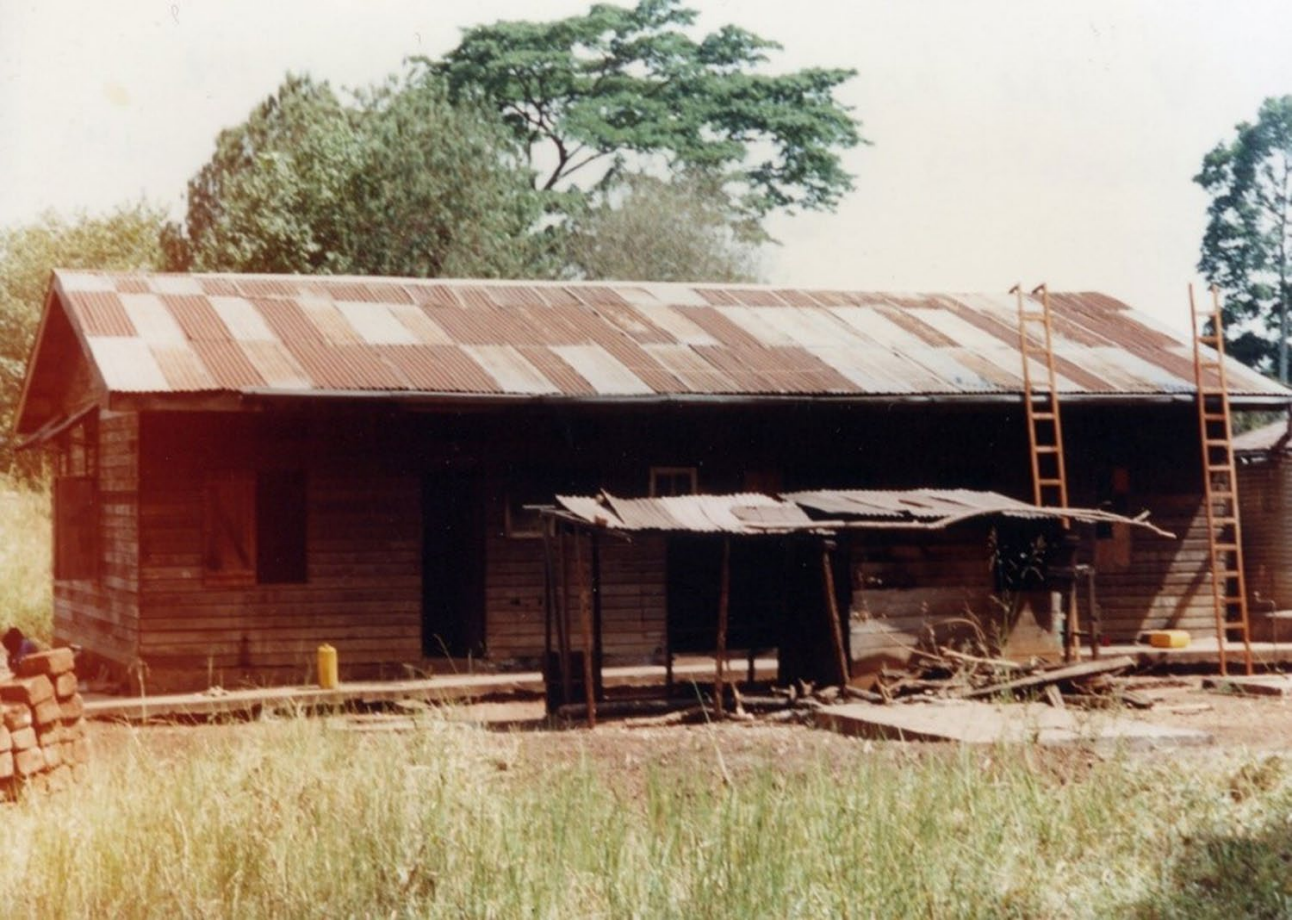
Project headquarters as we found them in 1991

On my next trip, in the autumn of 1990, Chris met me at the airport in Entebbe and told me he had a surprise for me. He told me that he had relocated the Budongo Forest Project to a new site in the middle of the forest at the site of the abandoned sawmill. We travelled to Budongo and drove past the forestry college, into the forest, to the new site of our project. I was dismayed to see the ruined houses that were to be our base. However, Chris had made contact with a group of builders and was ready to engage them to make a start on renovating the old ruined houses, and to build new accommodation for our staff (Fig. 3). At that time our field assistants preferred to live on-site and needed accommodation. The trail cutters, however, did not want to live on-site, and came in each day from their homes in surrounding villages. Chris had also engaged a cook-housekeeper, who lived on site.

Habituation of the chimpanzees and construction of the trail system, together with renovation of the houses, were the main activities during the early years. Slowly the chimpanzees became used to human presence and learned that there was nothing to fear from our field assistants. The adult males were the first to tolerate human presence. After them came the younger males and females, and lastly the mothers who had young infants. It was 5 years before we had habituated all the members of our community, which we called the Sonso community after the local river that runs not far from our camp and to this day supplies us with water in the dry season.

Besides habituation and camp construction, during these early years we had a succession of university students and senior researchers coming out to Budongo, mainly from the UK but also from other countries. They wrote M.Sc. dissertations, Ph.D. theses, reports, and publications. We kept careful records of all of these and set up a library at camp to house them.

In 1991 we were joined by Andrew Plumptre. Andy had already completed his Ph.D. on gorilla ecology, and came to us to study not just the chimpanzees but also the ecology of the forest and its wildlife. After learning to identify the forest tree species, he surveyed their distribution across the forest and discovered that the youngest part of the forest was in the southwest and the oldest part in the northeast. Young forest in Budongo is characterised by colonising species such as *Maesopsis eminii.* Young forest is then followed in the succession by mixed forest, which includes fig and mahogany species, and this in turn is followed by mature forest, which is characterised at Budongo by the climax tree species *Cynometra alexandri*, the ironwood (Eggeling 1947). Andy’s research focused on the effects of logging on the composition of the forest and its wildlife (Plumptre 1996; Plumptre and Reynolds 1994).

Andy was with us from 1991 to 1997. During the 1990s we appointed Fred Babweteera as director of the Budongo Forest Project, which established itself as a centre of excellence in wildlife and forestry research and gained something of an international reputation. In 1997, core funding from the British government ended and was taken over by the Norwegian Agency for Development Cooperation (NORAD), the Norwegian government’s development agency. We continued with research on chimpanzees and their role in the forest’s ecology, and researchers came and went. Some studied the three species of forest monkeys found in Budongo Forest (blue monkeys, redtail monkeys and black and white colobus monkeys), while others made studies of the forest birds, rodents and amphibians. The names of researchers and the titles of publications up to 2004 can be found in Reynolds (2005).

## The station becomes a non-governmental organisation

In 2007 the Budongo Forest Project changed its name to the Budongo Conservation Field Station (BCFS). This came about as a result of our acceptance by the Ugandan government as a registered Ugandan non-governmental organisation (NGO). This was Fred Babweteera’s achievement, and put us properly on the map of Uganda’s institutions, something we had always wanted. During the negotiations, Fred was informed that a “project” could not be an NGO, whereas a “field station” could. Hence the name change.

In addition to our studies on the forest and its wildlife, we now increased our involvement with people living in the local villages. This community focus was important for our conservation work, and we extended it as time went by. As has come to be understood in recent years, the main guardians of the world’s forests and wildlife are the people who live in and around those forests. Without their active participation, forest destruction and the loss of forest wildlife cannot be slowed down or stopped. From the start of our project, some of our researchers worked in the nearby village of Nyabyeya and studied the use of forest products (Johnson 1993) and the types of employment available for local people (Lauridsen 1999). We now began to focus more on raising conservation awareness in local villages, and in due course our vet took on the role of conservation co-ordinator.

## Conservation and threats to chimpanzees

**Fig. 4:**
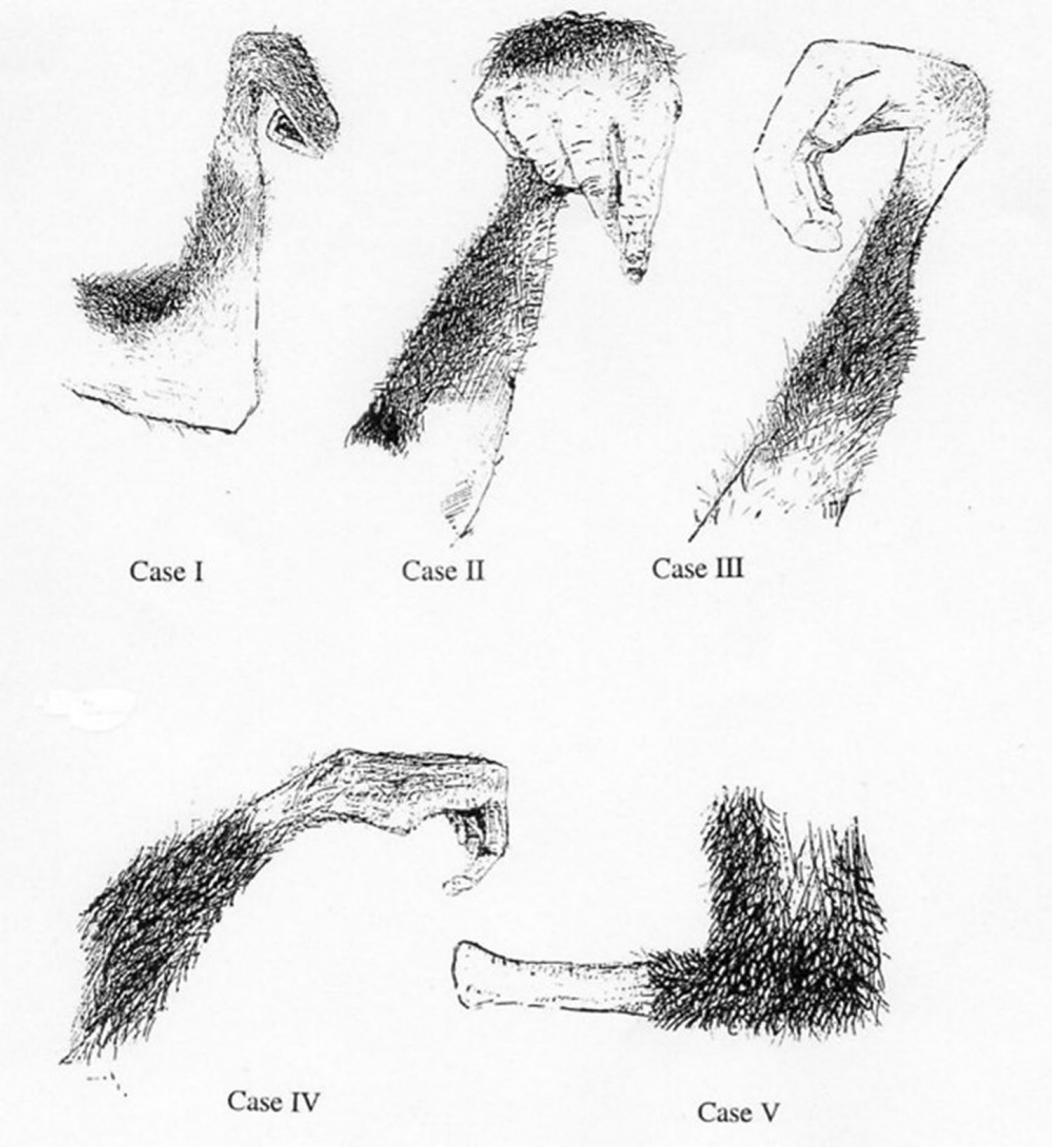
Snare injuries of chimpanzees (see Waller and Reynolds 2001)

Conservation awareness raising, in itself, did not bring any practical benefits to the farmers living in the local community, and that is what we then set out to put right. The majority of the households in the villages around Budongo are subsistence farmers, that is to say they only grow food for themselves and their families; they do this in fields, known as *shambas*, around their houses. One of our first projects was to build a borehole for the local villagers. This was made possible by a budget line for community assistance in the NORAD grant. In due course we also made improvements to the local schools by providing new latrines, and we made nine new floors for the classrooms in the large primary school at Nyabyeya. We also went into local schools to teach children the basic principles of conservation, explaining that chimpanzees are an endangered species in need of our protection. We showed them wire snares that we had removed from the forest, and we explained that although snares were put in the forest to catch duikers and pigs, they also unfortunately caught chimpanzees. We showed them pictures of chimpanzees that had put their hands into snares and had been unable to get away without losing the use of a hand or, in other cases, a foot (Fig. 4).

We hoped that the message we were giving the children would in due course find its way to their parents, and that appeared to have happened, as we got reports from members of our staff of the growing awareness among village people of the damage their snares were doing to our chimpanzees. They had not previously realised that chimpanzees were suffering as a result of getting caught in snares and then wrenching themselves free.

We employed four (later six) snare removers whose job was to go into the forest each day, find snares and remove them. This was practical conservation work. Our snare removers were ex-hunters: men who had up to then been putting snares in the forest but who had decided not to do so any more in view of the adverse effects on chimpanzees, as well as the alternative income opportunities that the Budongo project provided. We thought they would probably suffer some hostility from other members of their village who wanted to continue putting snares in the forest. However, to our surprise, that did not happen.

**Fig. 5:**
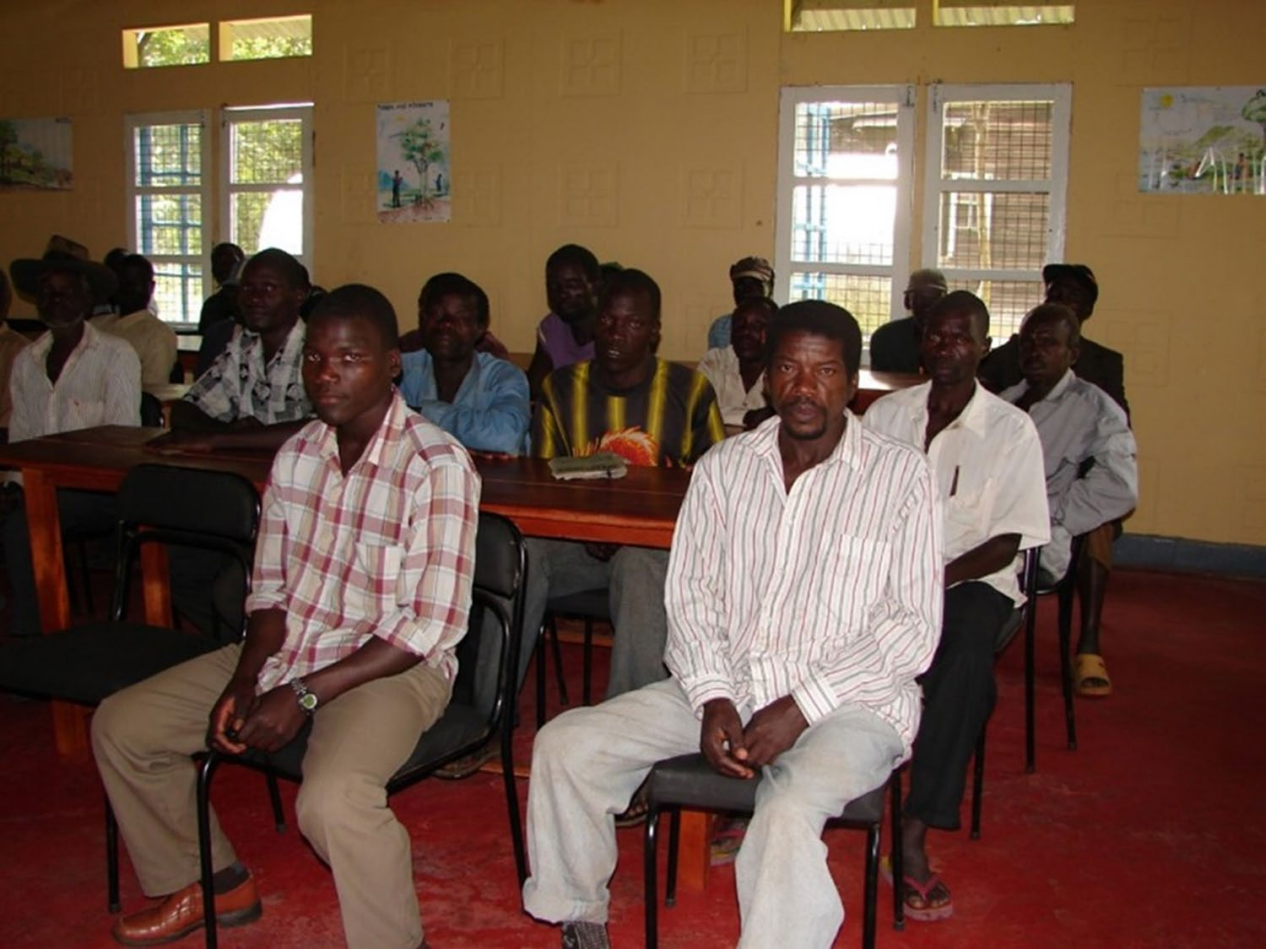
Conservation meeting for village farmers

Despite the fact that we were removing many snares from the forest, our chimpanzees continued to get caught and lose the function of a hand or a foot. Our director Fred Babweteera conceived the idea of an intervention that came to be known as our “goat scheme”. We called the local village men to a meeting at our camp (Fig. 5) where we put the idea to them that if they were willing to forgo putting snares in the forest we would provide them free of charge with two female goats, and we would bring round a male goat so that they would then be able to start a goat herd. Goat meat is highly regarded in Uganda and is an excellent source of protein for local people, but because of the difficulties of keeping goats, the prevalence of various diseases, and the cost, not many families were keeping goats at that time.

The men, local farmers who did some hunting on the side, listened carefully to our suggestion and went away to consider it and discuss it among themselves. They returned with the objection that the goats would succumb to disease. We responded by saying that our vet would come round monthly to check on the health of their goats and if any of them died we would replace them. We made it clear that if anyone joined the scheme and was subsequently found to be setting snares, he would lose his goats and would not be allowed to rejoin the scheme.

## Alternative livelihoods

**Fig. 6:**
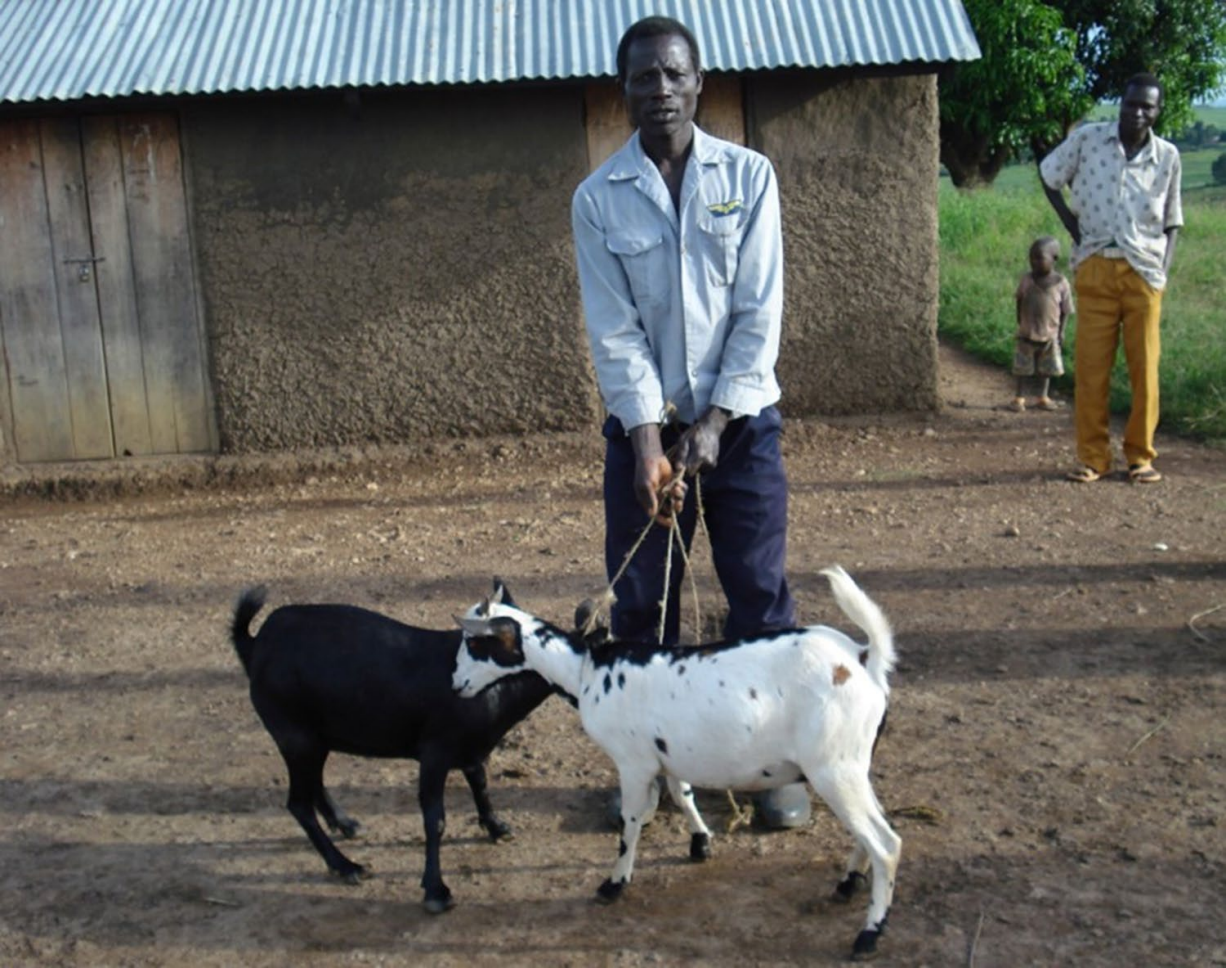
Local farmer with goats provided by Budongo Conservation Field Station

This project proved successful. The number of snares in the forest went down in our local area. However, when our snare removers extended their work further into the forest, we found that snares were still being set further from camp by people in other villages. As a result we extended the scheme further afield, and it continues forest-wide to the present day (Fig. 6).

In today’s world it is not sufficient for field projects to study primates or other species without getting involved in local conservation work. Because of the continuing decline in numbers of chimpanzees, indeed of all primates and other wildlife species, there is now a greater need than ever for all of us who work in the field, whether in Africa, Asia or South America, to become involved in finding ways and means of protecting the species we study. This does not mean a reduction in research, far from it. Research is needed as never before to determine the ranges and other necessities for life of the species we study.

Surveys are an important component of conservation and they need to be done carefully and with knowledge of the pitfalls awaiting the unwary. Andy Plumptre conducted a high-quality survey of the chimpanzees and forest monkeys in Budongo Forest in the mid-1990s, and this was repeated at 5-year intervals (Plumptre et al. 2010). Andy left us in 1997, but in his new job with the Wildlife Conservation Society he was able to extend his survey work along the whole of the Albertine Rift.

In 2005, Prof. Klaus Zuberbühler joined us as research director. He had a special interest in the vocalisations of chimpanzees, and his research and that of his students has since largely focused on this intriguing problem. In our first study of the Budongo chimpanzees in 1962, we focused on the chorusing of several chimpanzees together, later to be called pant-hoot chorusing. Subsequent research has shown that chimpanzees have a wide range of calls besides their loud chorusing. Spectrographic analysis of calls, their structure, the context in which they are given, the reactions of others and the status of who is calling, are some of the topics studied in this research area, and there is still much to find out (Zuberbühler 2019,2020). A related strand of research concerns gestures and their meaning as determined by the reactions of others. This work has been spearheaded by Catherine Hobaiter (Hobaiter et al. 2017), who has also pioneered the use of citizen science in primatology in connection with her comparative work on chimpanzee and human gestures.

## We employ a veterinarian

**Fig. 7:**
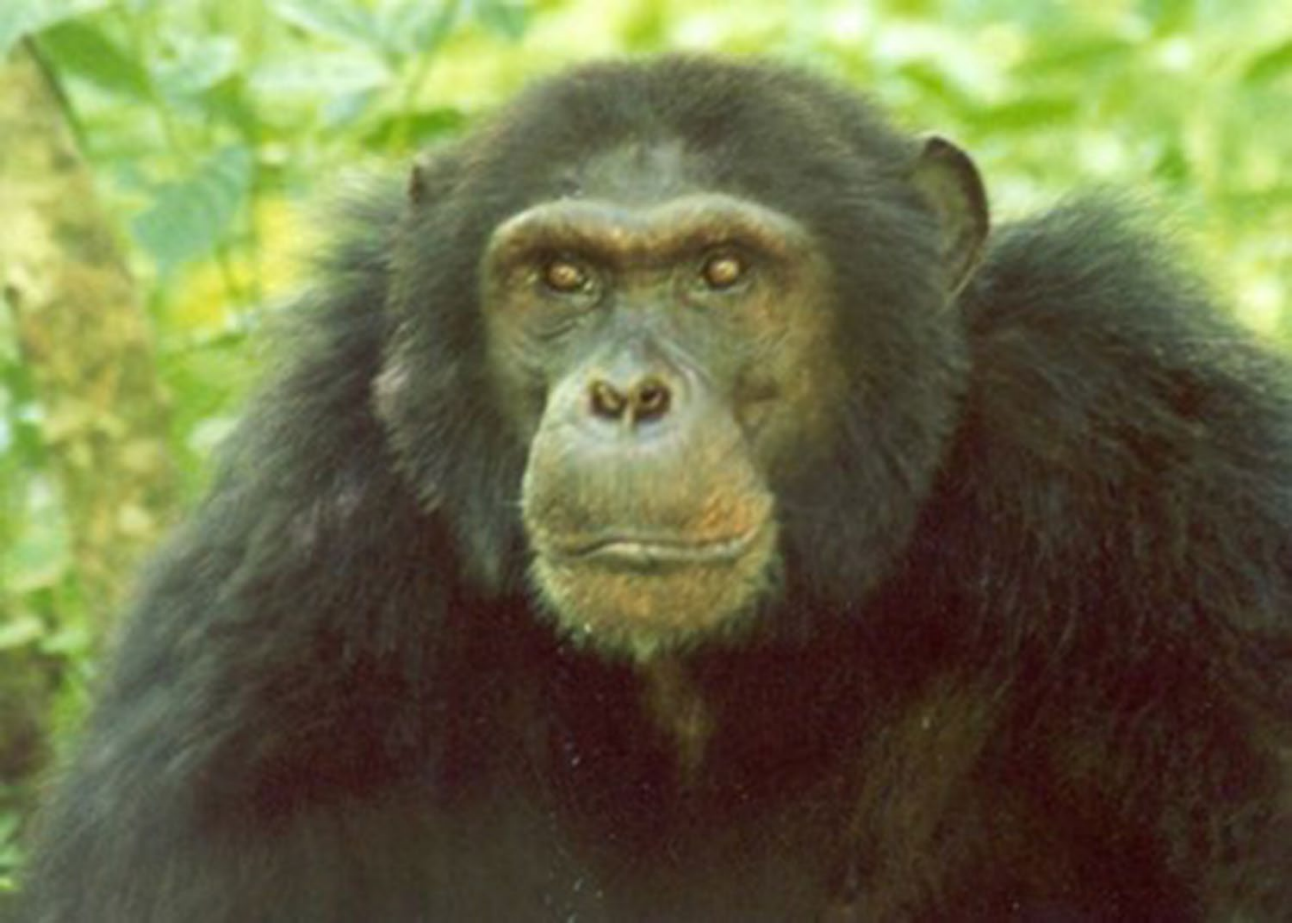
Alpha male Duane

A particular event led us to the decision to employ a wildlife vet on a permanent basis. This was the sudden death of our alpha male Duane in February 2008. He was an active leading male in the Sonso community when he suddenly died (Fig. 7).

Earlier there had been an outbreak of Ebola in West Africa, which had killed a number of chimpanzees, including adult males (Formenty et al. 1999). We were concerned that Duane might have died of Ebola or some other serious and possibly infectious disease. These fears were unfounded, but we realised we had no veterinary expertise at Budongo. We put the need for a vet to the Royal Zoological Society of Scotland, which had taken over our core funding in 2005 when NORAD support ended. The zoo’s director, David Windmill, immediately understood this need and agreed to provide funding for a vet. We advertised the new position and eventually hired Caroline Asiimwe, an excellent Ugandan vet. She much improved our veterinary work and extended it to several other forests in Uganda where chimpanzees were being studied. Thus Budongo Conservation Field Station became a national centre for chimpanzee health monitoring, and this is still the situation at the present time. We set up a laboratory and equipment on site to identify parasites from faecal samples. This field laboratory at BCFS has offered internship opportunities to a series of graduates from the Makerere University Veterinary School in Kampala.

With Klaus now responsible for research I realised that I was now, for the first time, free to undertake a research project of my own. Since the mid-1990s we had observed the Budongo chimpanzees doing something remarkable: making holes in the bottom of dead trees of the species *Raphia farinifera*. This species of raffia palm tree grew in swamp forest where the River Sonso regularly overflowed its banks and the forest floor became flooded. Raffia palms were, during the 1990s, quite common along the banks of the river in areas that flooded.

**Fig. 8:**
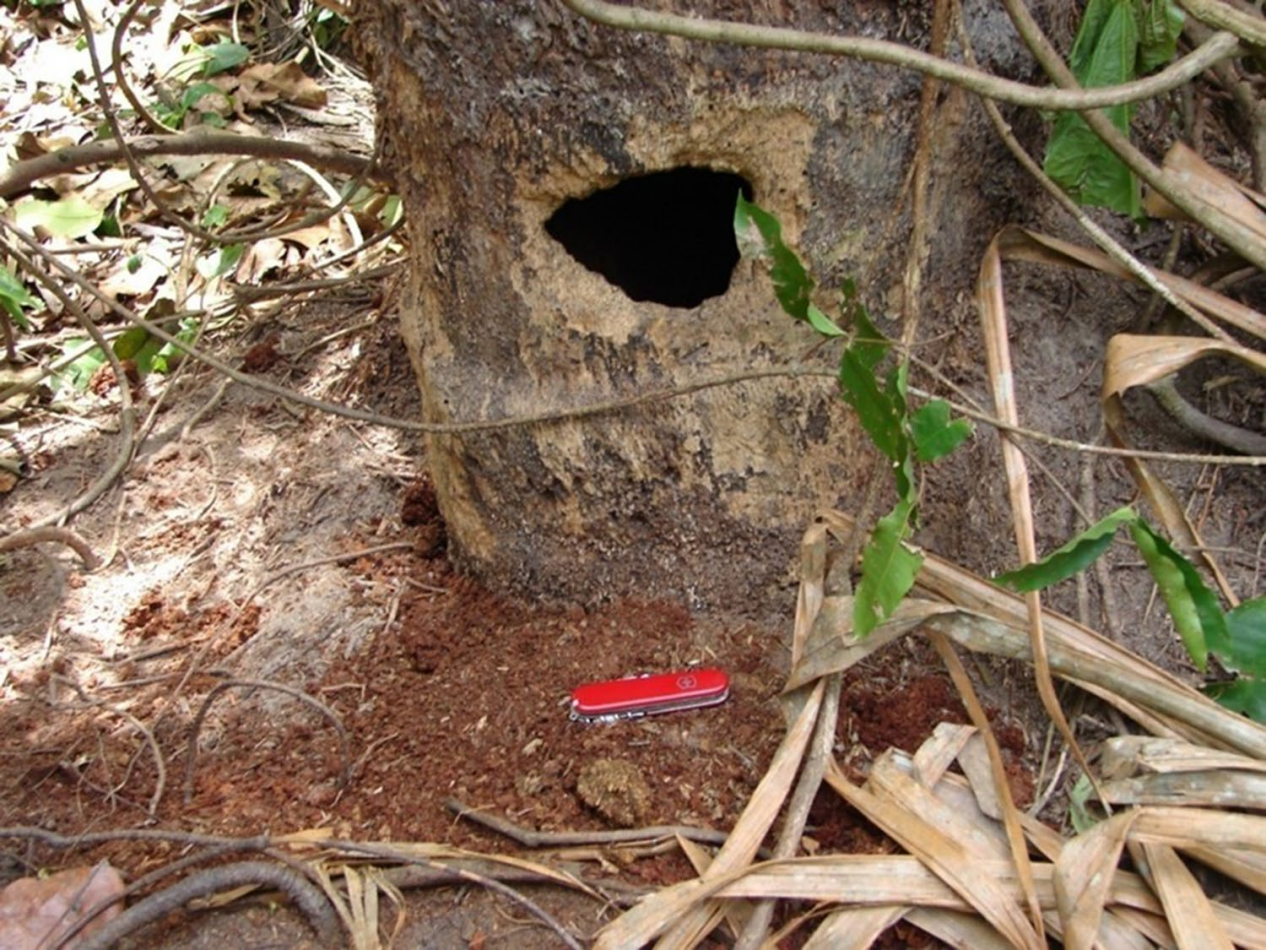
Base of dead* Raphia farinifera* with feeding hole made by chimpanzees. Note the bolus or “wadge” in front of the pocket knife

It was when walking through the swamps that we would find dead raffia trees with a smaller or larger hole in one side of the trunk near the bottom (Fig. 8). And sometimes we found a chimpanzee sitting beside the tree, putting a hand into the hole, pulling out dead pithy wood from the inside of the trunk, chewing it for some time and then spitting it out in the form of a “wadge” of unwanted pith. It seemed that the chimpanzees were extracting something from the decaying pith of these dead raffia palm trees, but we did not know what it was.

## Research on dietary minerals

In 2006 a paper appeared written by Jessica Rothman and co-workers (Rothman et al. 2006) in which they described the eating of dead wood by gorillas. Rothman et al. (2006) showed that the dead wood contained a high level of sodium, and it seemed possible that our chimpanzees might be extracting sodium by eating the pith of *R. farinifera*. I therefore looked for a lab that could do an elemental analysis for us, to see if there was any sodium in the raffia pith. I approached a colleague of mine, Andrew Lloyd, who was dean of science at the University of Brighton, about this. He responded positively and offered to analyse our Budongo raffia pith for its mineral content.

The first analysis gave us our answer. We collected and dried a variety of fruits, flowers and leaves that the Sonso chimpanzees ate, and samples of raffia pith, and Andrew’s lab put them through an elemental analyser. The results were clear: the pith contained a high level of sodium, probably in the form of sodium chloride or another sodium salt, in an otherwise sodium-poor environment. The findings were published in Reynolds et al. (2009) and were a nice complement to Rothman et al.’s (2006) paper, and a new discovery for chimpanzees. I followed this work with a series of further studies focusing on the various minerals ingested by the Budongo chimpanzees (Reynolds et al. 2012, 2015, 2019).

This brings us to the present time. In the last few years the Budongo Forest has faced a series of new threats. There have been threats in the past, for example from over-exploitation of *Cordia millenii* trees for boat building. The fruit and flowers of *C. millenii* are favourite foods of the chimpanzees, but the trees have now all but gone. The same has happened to the *R. farinifera* (raffia) palm trees. Their long leaves have been removed by tobacco farmers, who use the raffia twine to hang the tobacco leaves up in their curing houses. The trees have now mostly gone, but BCFS has replanted 2000 seedlings in the research area in the hope that some will survive. With the disappearance of *R. farinifera* the chimpanzees have lost this valuable source of sodium.

## Oil: the new threat

Today there is a new threat to the wildlife of Budongo Forest: oil. This is a new kind of threat, as it is on a completely different scale. Many millions of dollars have been spent, and continue to be spent, on oil extraction along the eastern shore of Lake Albert, which lies a mere 30 km to the west of Budongo, in the Albertine Rift. Two new oil roads, 30 feet wide, raised, and tarmacked for fast heavy oil tankers, have already been built through Budongo Forest, though fortunately not through the middle of the forest where our camp is located. One cuts through the northwest part and the other through the southeast part of the forest. Apart from being a blight on the landscape, these oil roads herald a new era in Budongo’s history. As I write, the oil is not yet flowing, but in a few years’ time it will. More people will then come to the area to find work. New houses and villages will be constructed. Pressure to find firewood will bring people into the forest in larger numbers than before. Snaring will increase and more chimpanzees will be injured. Human diseases against which the chimpanzees have no immunity, such as the common cold, will come into the forest.

Other primate projects in other parts of Africa and in South America and Asia have faced similar threats, from mining, oil extraction, clear-felling for agriculture (for soya and palm oil) and for pasture for livestock. Such developments, due to the ever-expanding human population across the world, have led to the decimation or extinction of wildlife, and many primate species are at risk. We can but hope that governments and industries will take steps to preserve what is left of the world’s forests, but there is not much scope for optimism given what is happening today.

For this reason, in my address to the IPS Congress held at Quito in 2022, I argued for the creation of a network of primate field stations, so that they can join forces to better mitigate the pressures due to the activities of large-scale companies and industries. I used an analogy taken from Bushman folklore: Can an ant defeat an elephant? No, it cannot. But an army of ants can. Let us hope that the large and wonderful variety of non-human primate species that still inhabit the wild areas of the world today will be able to survive into the future. They will need our help to do so.

### Advice to upcoming primatologists

One of the reviewers of this paper suggested that upcoming primatologists might benefit from any advice I could give about field primatology and research. Advice is always a risky business because, it is said, “Never give advice because a wise man doesn’t need it and a fool won’t take it”. Having said that, I think there are certain things that make for successful field primatology. First of all, you need to have a high level of motivation in order to put up with all sorts of things going wrong, from bureaucratic difficulties, such as getting permits, to insect bites in places you’d rather not talk about. I have said to students, “Assume that things will go wrong”, and things have gone wrong. A second important aspect of fieldwork is to make a pilot study. Try to work out, given the situation you find yourself in, the best methods to get results, and the amount of work that will be needed. Work out how long it will take and multiply it by two. With plenty of motivation and a clear idea of what you are trying to do and how to do it, you’ll be okay.
